# Post-stroke Rehabilitation Training with a Motor-Imagery-Based Brain-Computer Interface (BCI)-Controlled Hand Exoskeleton: A Randomized Controlled Multicenter Trial

**DOI:** 10.3389/fnins.2017.00400

**Published:** 2017-07-20

**Authors:** Alexander A. Frolov, Olesya Mokienko, Roman Lyukmanov, Elena Biryukova, Sergey Kotov, Lydia Turbina, Georgy Nadareyshvily, Yulia Bushkova

**Affiliations:** ^1^Research Institute of Translational Medicine, Pirogov Russian National Research Medical University Moscow, Russia; ^2^Laboratory of Mathematical Neurobiology of Learning of Institute of Higher Nervous Activity and Neurophysiology, Russian Academy of Sciences Moscow, Russia; ^3^Department of Neurorehabilitation and Physiotherapy of Research Center of Neurology, Russian Academy of Medical Sciences Moscow, Russia; ^4^Department of Neurology, Vladimirsky Moscow Regional Research Clinical Institute Moscow, Russia; ^5^Medical Faculty, Pirogov Russian National Research Medical University Moscow, Russia; ^6^Research Institute of Cerebrovascular Pathology and Stroke, Pirogov Russian National Research Medical University Moscow, Russia

**Keywords:** ClinicalTrials.gov, identifier: NCT02325947, brain-computer interface, motor imagery, rehabilitation, stroke, paresis, exoskeleton

## Abstract

Repeated use of brain-computer interfaces (BCIs) providing contingent sensory feedback of brain activity was recently proposed as a rehabilitation approach to restore motor function after stroke or spinal cord lesions. However, there are only a few clinical studies that investigate feasibility and effectiveness of such an approach. Here we report on a placebo-controlled, multicenter clinical trial that investigated whether stroke survivors with severe upper limb (UL) paralysis benefit from 10 BCI training sessions each lasting up to 40 min. A total of 74 patients participated: median time since stroke is 8 months, 25 and 75% quartiles [3.0; 13.0]; median severity of UL paralysis is 4.5 points [0.0; 30.0] as measured by the Action Research Arm Test, ARAT, and 19.5 points [11.0; 40.0] as measured by the Fugl-Meyer Motor Assessment, FMMA. Patients in the BCI group (*n* = 55) performed motor imagery of opening their affected hand. Motor imagery-related brain electroencephalographic activity was translated into contingent hand exoskeleton-driven opening movements of the affected hand. In a control group (*n* = 19), hand exoskeleton-driven opening movements of the affected hand were independent of brain electroencephalographic activity. Evaluation of the UL clinical assessments indicated that both groups improved, but only the BCI group showed an improvement in the ARAT's grasp score from 0 [0.0; 14.0] to 3.0 [0.0; 15.0] points (*p* < 0.01) and pinch scores from 0.0 [0.0; 7.0] to 1.0 [0.0; 12.0] points (*p* < 0.01). Upon training completion, 21.8% and 36.4% of the patients in the BCI group improved their ARAT and FMMA scores respectively. The corresponding numbers for the control group were 5.1% (ARAT) and 15.8% (FMMA). These results suggests that adding BCI control to exoskeleton-assisted physical therapy can improve post-stroke rehabilitation outcomes. Both maximum and mean values of the percentage of successfully decoded imagery-related EEG activity, were higher than chance level. A correlation between the classification accuracy and the improvement in the upper extremity function was found. An improvement of motor function was found for patients with different duration, severity and location of the stroke.

## Introduction

Motor imagery (Page et al., 2001), or mental practice, attracted considerable interest as a potential neurorehabilitation technique improving motor recovery following stroke (Jackson et al., 2001). According to the Guidelines for adult stroke rehabilitation and recovery (Winstein et al., 2016), mental practice may proof beneficial as an adjunct to upper extremity rehabilitation services (Winstein et al., 2016). Several studies suggest that motor imagery can trigger neuroplasticity in ipsilesional motor cortical areas despite severe paralysis after stroke (Grosse-Wentrup et al., 2011; Shih et al., 2012; Mokienko et al., 2013b; Soekadar et al., 2015).

The effect of motor imagery on motor function and neuroplasticity has been demonstrated in numerous neurophysiological studies in healthy subjects. Motor imagery has been shown to activate the primary motor cortex (M1) and brain structures involved in planning and control of voluntary movements (Shih et al., 2012; Mokienko et al., 2013a,b; Frolov et al., 2014). For example, it was shown that motor imagery of fist clenching reduces the excitation threshold of motor evoked potentials (MEP) elicited by transcranial magnetic stimulation (TMS) delivered to M1 (Mokienko et al., 2013b).

As motor imagery results in specific modulations of brain electroencephalographic (EEG) signals, e.g., sensorimotor rhythms (SMR) (Pfurtscheller and Aranibar, 1979), it can be used to voluntarily control an external device, e.g., a robot or exoskeleton using a brain-computer interface (BCI) (Nicolas-Alonso and Gomez-Gil, 2012). Such system allowing for voluntary control of an exoskeleton moving a paralyzed limb can be used as an assistive device restoring lost function (Maciejasz et al., 2014). Besides receiving visual feedback, the user receives haptic and kinesthetic feedback which is contingent upon the imagination of a specific movement.

Several BCI studies involving this type of haptic and kinesthetic feedback have demonstrated improvements in clinical parameters of post-stroke motor recovery (Ramos-Murguialday et al., 2013; Ang et al., 2014, 2015; Ono et al., 2014). The number of subjects with post-stroke upper extremity paresis included in these studies was, however, relatively low [from 12 (Ono et al., 2014) to 32 (Ramos-Murguialday et al., 2013) patients]. As BCI-driven external devices, a haptic knob (Ang et al., 2014), MIT-Manus (Ang et al., 2015), or a custom-made orthotic device (Ramos-Murguialday et al., 2013; Ono et al., 2014) were used. Furthermore, several other studies reported on using BCI-driven exoskeletons in patients with post-stroke hand paresis (Biryukova et al., 2016; Kotov et al., 2016; Mokienko et al., 2016), but these reports did not test for clinical efficacy and did not include a control group. While very promising, it still remains unclear whether BCI training is an effective tool to facilitate motor recovery after stroke or other lesions of the central nervous system (CNS) (Teo and Chew, 2014).

Here we report a randomized and controlled multicenter study investigating whether 10 sessions of BCI-controlled hand-exoskeleton active training after subacute and chronic stroke yields a better clinical outcome than 10 sessions in which hand-exoskeleton induced passive movements were not controlled by motor imagery-related modulations of brain activity. Besides assessing the effect of BCI training on clinical scores such as the ARAT and FMMA, we tested whether improvements in the upper extremity function correlates with the patient's ability to generate motor imagery-related modulations of EEG activity.

## Materials and methods

### Study design

This randomized, blind, controlled study was conducted at three medical centers from December, 2014 to August, 2016. The center selection criteria included the presence of a neurorehabilitation department or motor rehabilitation service and availability of patients with different post-stroke periods and with hemiparesis of different severity.

The inclusion criteria were as follows: male or female patients which underwent inpatient treatment at the study centers, aged from 18 to 80 years, with subacute (1–6 months from onset) or chronic (>6 months from onset) stroke; hand paresis, mild to plegia, according to the Medical Research Council Sum-Score scale (Compston, 2010); a single focus of ischemic or hemorrhagic stroke with a supratentorial localization (according to MRI or CT data); and a signed informed consent. Such a heterogeneous group was chosen in order to find a target group of patients for which the BCI + hand exoskeleton procedure is the most efficient.

The exclusion criteria were as follows: left-handedness according to the Edinburgh Handedness Inventory (Oldfield, 1971); severe cognitive impairment (<10 points according to the Montreal Cognitive Assessment Scale; Bocti et al., 2013); sensory aphasia; severe motor aphasia; severe vision impairment preventing execution of visual instructions shown on the computer screen; muscle spasticity in the upper extremity 4 points according to the Modified Ashworth Scale (mAS 1–5 points; Bohannon and Smith, 1987).

The withdrawal criteria were as follows: patient refusal to continue participating in the study; development of an acute disease or decompensation of a chronic disease with the risk of a potential impact on the study results (repeated stroke, acute myocardial infarction, non-compensated diabetes, etc.); prescription of systemic muscle relaxants or changing their dose after inclusion to the study; injection of botulinum toxin agents in muscles of the paretic upper extremity after inclusion of the patient to the study.

**Figure 1:**
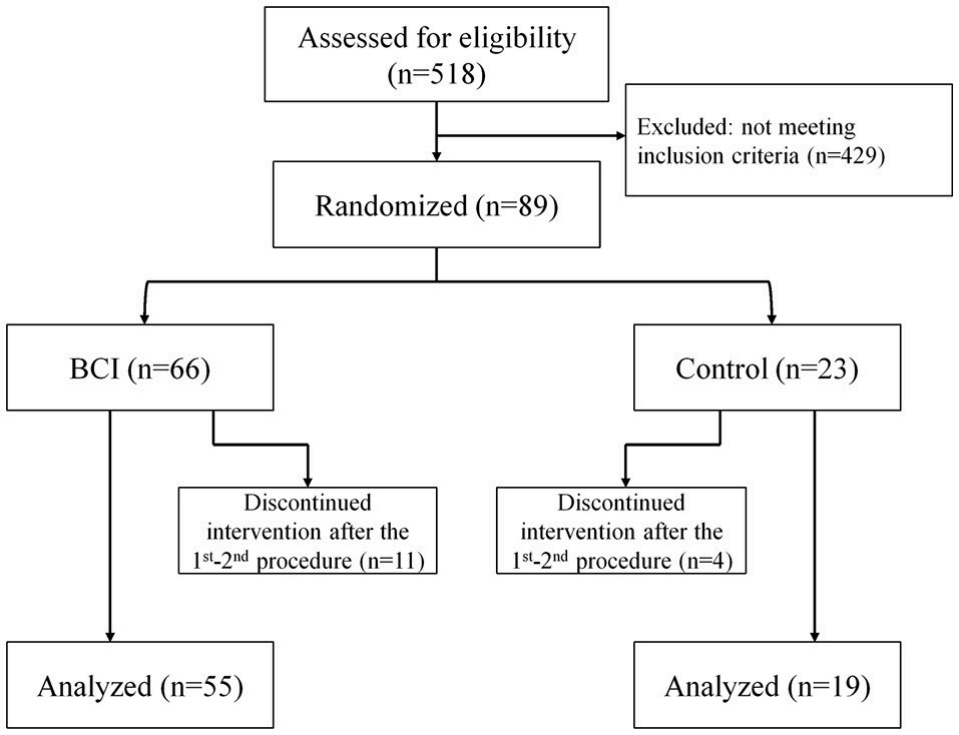
The subject flow diagram from recruitment through analysis (Consolidated Standards of Reporting Trials flow diagram).

A total of 518 patients were screened for participation in the study. Eighty-nine patients met the inclusion criteria; 15 of them refused to participate in the study after the first or second intervention. The study included 74 patients; 48 males; the median age was 58 [50.0; 65.0] years. The subject flow diagram from the recruitment through the analysis is presented in Figure 1. Fifty-five patients had upper extremity paresis due to ischemic stroke and 19 patients due to hemorrhagic stroke. The lesion location was cortical in 4 cases, subcortical in 41 cases and cortico-subcortical in 29 cases. The median stroke duration was 8.0 [3.0; 13.0] months, upper extremity paresis severity was 4.0 [0.0; 30.0] points by ARAT and 19.5 [11.0; 40.0] points by FMMA.

The examination data of patients who signed the informed consent and met the inclusion criteria were uploaded to an automated system for clinical research information support (Imagery Soft, Russia). The system assigned an identification number (ID) to each study participant. The IDs were randomized: ¾ of the patients were assigned to the BCI group, and ¼ was assigned to the control group. Thus, the BCI and control groups consisted 55 and 19 patients, respectively. The reduced number of patients in the control group compared with the BCI group is the result of compromise between the intention to increase the number of patient undergoing the intensive motor imagination training and a sufficiently high statistical power of the study. Main demographic and baseline data of patients of each group are shown in Table 1. The groups were matched by age, stroke duration, type of stroke, lesion localization, and ARAT or FMMA scores according to Mann–Witney criterion (*p* > 0.05). Since the BCI and control groups were equivalent in the above parameters, we suggest that the net effect of motor recovery in both groups depends only on the difference between active and passive conditions. Table 2 represents the baseline ARAT and FMMA scores for patients of different stroke period within each study group.

**Table 1:** Demographics and main baseline characteristics of subjects by study group.

**Variable**	**BCI (*n* = 55)**	**Control (*n* = 19)**	***p* Mann–Witney**
			**criterion**
Age, years	58.0 [48.0; 65.0]	58.0 [52.0; 67.0]	0.581
Males, % (n)	61.8 (34)	73.7% (14)	
Time from stroke onset, months	8.0 [4.0; 13.0]	8.0 [1.0; 13.0]	0.515
**STROKE PERIOD, % (N)**			
Subacute (1–6 months from onset)	45.5 (25)	47.4 (9)	
Chronic (>6 months from onset)	54.5 (30)	52.6 (10)	
**LESION LATERALIZATION, % (N)**			
Left hemisphere	47.3 (26)	63.2 (12)	
Right hemisphere	52.7 (29)	36.8 (7)	
**LESION LOCALIZATION, % (N)**			
Cortical	3.6 (2)	10.5 (2)	
Subcortical	58.2 (32)	47.4 (9)	
Corticosubcortical	38.2 (21)	42.1 (8)	
Initial ARAT score	4.0 [0.0; 31.0]	3.0 [0.0; 30.0]	0.722
Initial FMMA upper extremity score	24.0 [12.0; 40.0]	12.0 [11.0; 49.0]	0.363
Initial spasticity (mAS)	3.0 [1.0; 4.0]	3.0 [2.0; 4.0]	0.732

*Medians and 25 and 75% quartiles are shown*.

**Table 2:** Baseline ARAT and FMMA scores by study group and stroke period.

**Outcome**	**Study group**		***p* Mann–Witney criterion**
	**BCI**	**Control**	
**SUBACUTE PATIENTS (1–6 MONTHS FROM ONSET)**			
***n***	**25**	**9**	**n/a**
ARAT total	1.0 [0.0; 15.0]	13.0 [0.0; 22.0]	0.489
ARAT-Grasp	0.0 [0.0; 7.0]	4.0 [0.0; 7.0]	0.489
ARAT-Grip	0.0 [0.0; 4.0]	2.0 [0.0; 5.0]	0.565
ARAT-Pinch	0.0 [0.0; 1.0]	2.0 [0.0; 4.0]	0.335
ARAT-Gross movement	1.0 [0.0; 4.0]	2.0 [0.0; 5.0]	0.514
FMMA upper extremity	15.0 [6.0; 36.0]	12.0 [11.0; 29.0]	0.969
FMMA-Proximal	12.0 [7.0; 26.0]	11.0 [10.0; 20.0]	0.878
FMMA- Distal	2.0 [1.0; 9.0]	1.0 [1.0; 9.0]	0.591
**CHRONIC PATIENTS (**>**6 MONTHS FROM ONSET)**			
***n***	**30**	**10**	**n/a**
ARAT total	18.5 [1.0; 39.0]	2.0 [0.0; 30.0]	0.331
ARAT-Grasp	8.0 [0.0; 17.0]	0.0 [0.0; 12.0]	0.272
ARAT-Grip	4.5 [0.0; 9.0]	0.0 [0.0; 8.0]	0.432
ARAT-Pinch	2.0 [0.0; 10.0]	0.0 [0.0; 6.0]	0.379
ARAT-Gross movement	2.0 [1.0; 6.0]	2.0 [0.0; 6.0]	0.701
FMMA upper extremity	30.5 [17.0; 41.0]	12.5 [11.0; 49.0]	0.272
FMMA-Proximal	22.0 [15.0; 29.0]	11.5 [9.0; 27.0]	0.259
FMMA- Distal	8.0 [2.0; 15.0]	2.5 [1.0; 19.0]	0.569

*Medians and 25 and 75% quartiles are shown*.

Patients in the BCI group were trained with the BCI-controlled exoskeleton, whereas exoskeleton-driven hand movements in the control group were not linked to the patient's brain activity but following a repetitive scheme. The patients in each group performed 10 daily sessions. The session duration was 30 min. The sessions were conducted every day with breaks on weekends and holidays (up to 3 consecutive days). Patients in both groups were provided with standard physical therapy: instructor-supervised kinesiotherapy, medical massage, and passive neuromuscular electrical stimulation in accordance with Russian treatment protocols and standards.

### BCI protocol

**Figure 2:**
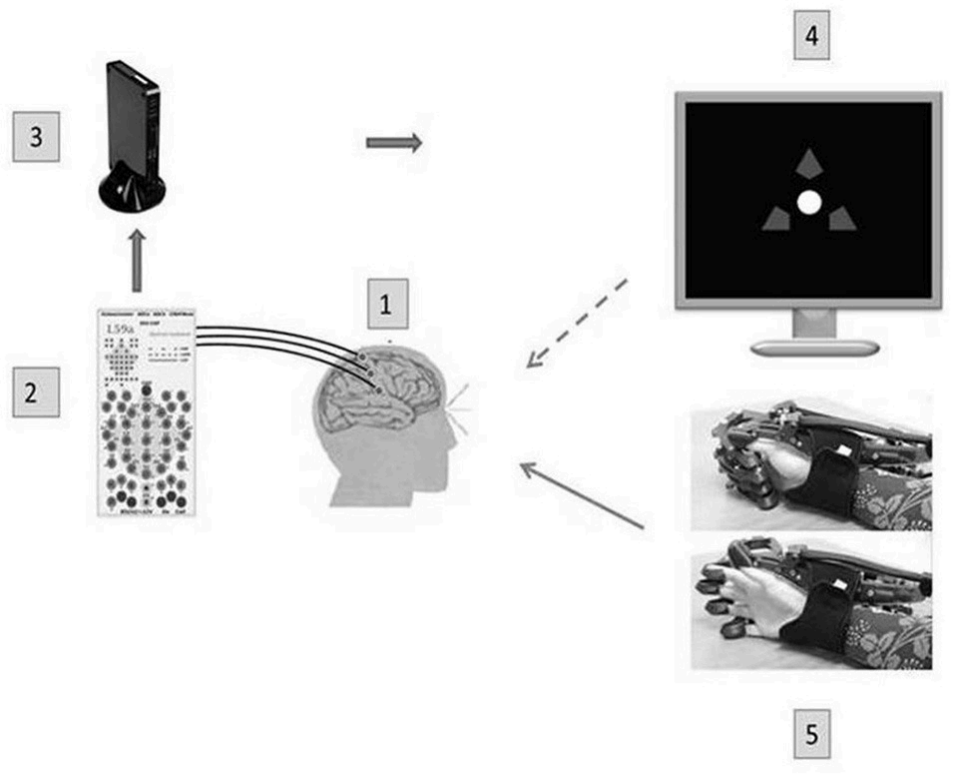
A BCI-exoskeleton complex. A block diagram of the BCI complex used in this study: 1—32 Ag/AgCl EEC electrodes, 2—a NVX 52 encephalograph (Medical Computer Systems, Russia); 3—a computer (OS Windows 7): real time data transmission, identification of operational EEG parameters, recognition of a steering instruction; 4—a presentation monitor; 5—a hand exoskeleton (Neurobotics, Russia) with pneumatic actuators of finger extensors and spring flexors; flexed and extended exoskeleton configurations are shown. The dotted arrow denotes a visual feedback, and the solid arrow denotes a kinesthetic feedback.

The design of the BCI-controlled exoskeleton is shown in Figure 2. The BCI was used to classify EEG patterns of three mental tasks: (1) motor relaxation, (2) imagery of left-hand opening, and (3) imagery of right-hand opening. Task instructions were provided using a computer screen. Assessing EEG brain activity during motor imagery of both the paretic and intact hand (instead of imagining the paretic hand moving only) allowed discrimination between both conditions. This requirement assured that patients performed a lateralized motor imagery and not a different mental task, for example a general increase of attention.

EEG signals were recorded with 30 electrodes placed according to the International 10–20 system (NVX52, Medical Computer Systems, Zelenograd, Russia): C1, C2, C3, C4, C5, C6, Cp1, Cp2, Cp3, Cp4, Cp5, Cp6, Cpz, Cz, F3, F4, Fc3, Fc4, Fc5, Fc6, Fcz, Fz, O1, O2, P1, P2, P3, P4, Po3, Po4, Poz, Pz with the reference electrode fixed randomly to the left or right earlobe. The sampling rate was 500 Hz. Data were filtered using a band-pass 5–30 Hz FIR filter (of order 101) and 50 Hz IIR notch Chebyshev type I filter (of order 6). The filters were designed using Matlab Filter Design Toolbox.

The previously implemented Bayesian classifier based on EEG covariance matrices calculated for the three mental tasks (Bobrov et al., 2012) was then applied. This algorithm slightly underperforms more sophisticated classifiers but outperforms in computational cost (Frolov et al., 2011). The classifier expressions are derived under the assumption that EEG has a multivariate Gaussian distribution with zero mean and covariance matrix depending on mental task performed. Therefore, in case a mental task is be to classified, EEG corresponding the *i*-th task performance is considered to be drawn from the distribution with probability density function

$$\begin{array}{l} p_{\iota }\left(X\right)=\frac{1}{\sqrt{{\left(2\pi \right)}^{N_{ch}}\cdot \text{det}\left(C_{\iota }\right)}}e^{-X^{T}C_{\iota }^{-1}X/2},\;\iota =1,\ldots ,\;N_{st} \end{array}$$

In the expression *N*_*ch*_ denotes the number of EEG channels, and *C*_ι_ is a signal covariance matrix corresponding to the ι-th state. It is assumed that during BCI control each mental task is required to be imagined with a probability equal to *1/N*_*st*_, i.e., that all mental states are equiprobable. According to Bayesian classification rule a signal sample *X* is considered to correspond to that state for which an a posteriori probability to correspond to the state is maximal, i.e.,

$$\begin{array}{l} c=\underset{i=1,\ldots ,N_{st}}{\text{argmax}}\frac{p_{\iota }\left(X\right)}{N_{st}} \end{array}$$

Here, maximizing an a posteriori probability corresponding to a certain state is equivalent to minimizing probability density function exponents with their sign reversed (1/2 multiplier is omitted as irrelevant for optimization)

$$\begin{array}{l} c=\underset{\iota =1,\ldots ,N_{st}}{\text{argmin}}\left(X^{\text{T}}C_{\iota }^{-1}X+\ln\;\left(\det\left(C_{\iota }\right)\right)\right)=\underset{\iota =1,\ldots ,N_{st}}{\text{argmin}}V_{\iota }\left(X\right) \end{array}$$

The expression for *V*_ι_(*X*) can be rewritten as

$$\begin{array}{l} V_{\iota }\left(X\right)=trace\left(XX^{T}C_{\iota }^{-1}\right)+ln\left(\det\left(C_{\iota }\right)\right) \end{array}$$

Estimates of mean values can be used by averaging over a selected EEG epoch. In this case, not a single sample but an epoch is classified by minimizing

$$\begin{array}{l} V_{\iota }=\text{trace}\left(CC_{\iota }^{-1}\right)+ln\left(\det\left(C_{\iota }\right)\right) \end{array}$$

where *C* denotes a signal covariance matrix estimate computed from the epoch data. It is easy to see that to train the Bayesian classifier means to estimate covariance matrices for EEG signals corresponding to different mental tasks. The classifier can adapt by changing these covariance matrix estimates. To sum up, the Bayesian classifier is adaptable, has extremely low computational cost, is able to classify an arbitrary number of states, and does not rely on any particular a priori frequency or spatial information. The last feature makes it an all-purpose classifier but may result in lower classification accuracy.

We did not control directly the amplitude of the sensorimotor rhythm, because, as was shown by preliminary experiments, its value and spatial localization are highly variable among patients.

The percentage of correctly classified trials was used as the indicator of BCI accuracy which depends on both the classifier performance and the participant's ability to perform motor imagery. We consider the confusion matrix as a general assessment of BCI accuracy. The percentages of trials in which paretic hand motor imagery-related EEG signal modulations, unaffected hand motor imagery-related EEG signal modulations and rest states were detected are the diagonal elements of this matrix. The average of these elements is used in the following as an index of BCI accuracy and, at the same time, of the ability to elicit motor imagery-related modulations of EEG signals. The chance level for classifying the different conditions correctly was 33%.

### BCI-exoskeleton-based training procedure

To record EEG activity, an EEG cap was placed on the patient's head; the EEG electrodes were filled with gel. The exoskeleton was attached to the paretic hand. The exoskeleton consisted of a polymeric movable frame that encased the hand and fingers, and pneumatic actuators that extended the fingers. During training, the patient sat at a table in front of a computer monitor with his arms on a table in front of him in a comfortable position.

Patients were instructed to fix their gaze on a white circle at the center of the computer screen. The screen background was black. The patient performed one of three mental tasks: (1) relax, (2) kinesthetic imagination of a continuous opening of the right hand, and (3) kinesthetic imagination of a continuous opening of the left hand. Recognition accuracy was evaluated for all three states.

The mental task instruction was provided by color changes of three arrows located around the circle which was visually presented on a computer screen. While relax/rest state was instructed by a green arrow on the top and white arrows on the left and right, motor imagery of left or right hand was instructed by a green arrow on the left or on the right, respectively, and a white arrow on the top. Instructions to imagine movements of the right or left hand were randomly selected and continued for 10 s. Each imagery period was followed by an instruction to relax/rest continued for 10 s also. A 10 s segment corresponding to one instruction constituted one trial. Trial length of 10 s is a compromise between a length that induces early tiredness/fatigue and a length that allows to record sufficient data for the classifier training (Frolov et al., 2012). Two instructions for motor imagery and two instructions to relax constituted one block of 40 s duration. Fifteen blocks constituted one session.

The classifier evaluated EEG signals every 100 ms over the recording of last 1 s. Thus, 100 evaluations were performed during presentation of one instruction. Visual and proprioceptive feedback signals changed every 100 ms. The classifier trained during the first block and then continued training after each block (Frolov et al., 2012). The duration of each session was 10 min with 5-min rest periods between the sessions. Each daily procedure consisted of three sessions and thus yielded 45 trials for the right-hand imagery and 45 trials for the left hand.

The patients received both visual and kinesthetic feedback of the results of BCI decoding of their imagery attempts. If the classifier successfully recognized the hand being imagined from the EEG patterns, the gaze fixation marker turned green, and the exoskeleton extended the fingers.

The speed of exoskeleton opening was proportional to the number of correct motor imagery recognitions during the last 1 s window. Since motor imagery-related EEG modulations were detected each 100 ms, the number of recognitions varied from 0 to 10. The extension was produced if this number exceed 3. Under maximal number of recognitions the exoskeleton entirely opened the fingers during 5 s.

In case when this number was less than 3 the marker's brightness reduced, and the exoskeleton flexed the fingers. The feedback was given online in a continuous way. Since the commands to exoskeleton opening with different speeds alternated with the commands to its closing, the exoskeleton could induce several flexions-extensions during one trial. No special means were used to hide the exoskeleton from patient view. During the rest periods patients sat quietly and looked at the screen center, the exoskeleton was closed and the feedback was switched off.

### Passive exoskeleton-driven movements of the paretic hand (sham condition)

In the control condition, we used the same arrangements as in the BCI sessions, including putting the EEG cap on the subject's head and fixing their paretic hands to the exoskeleton, but hand exoskeleton movements were not dependent on motor imagery-related EEG modulations. The patients performed the relaxation/rest task while watching for changes in the arrow colors. The right and left arrows were shown randomly. If the change in arrow color indicated movement of the paretic hand, the exoskeleton performed an opening movement.

Recordings of EEG activity for the control group as well as for the BCI group were to be used in future off-line analysis.

### Clinical assessment

Before and after the training sessions, the patients were assessed for movements and strength in the upper limb using the Fugl-Meyer Motor Assessment (FMMA) for upper extremity (range, 0–66) and Action Research Arm Test (ARAT; range, 0–57) (Sanford et al., 1993; Doussoulin et al., 2012). Additionally, the changes across different FMMA and ARAT sections were analyzed. The spasticity severity was assessed using the Modified Ashworth Scale.

We also estimated the percentage of patients with clinically significant improvement, or minimal clinically important difference (MCID) in each study group (BCI and control group) and subgroup (subacute and chronic). As recommended in the literature (Langhorne et al., 2011) MCID was chosen separately for subacute and chronic stroke. MCID for the ARAT scale is accepted to be 12 point increase for dominant and 17 point increase for non-dominant hand in case of subacute stroke and 6 point increase in case of chronic stroke (van der Lee et al., 2001a,b; Lang et al., 2008). MCID for the FMMA scale is accepted to be 10 point increase in case of subacute stroke and 5 point increase in case of chronic stroke (Shelton et al., 2001; Page et al., 2015).

### Blinding

The researchers who performed the clinical assessment of a patient with ARAT and FMMA were blinded, i.e., they were not aware to which of the two study groups the patients were assigned to. Information on the study group was available only to the researchers who performed the rehabilitation procedures. Each patient was examined before and after training sessions by the same assessor to avoid intra-rater scale variability. Three independent assessors from three clinical centers participated in the study which partially compensates a possible intra-rater bias in the assessments.

### Statistical methods

Statistical data analyses were performed using the independent sample *t*-test (sample size calculation), Mann–Whitney (comparison of independent samples), Wilcoxon (comparison of dependent samples) tests and Spearman correlation coefficient on a personal computer using a STATISTICA 6.0 application software package (StatSoft®, 2003). Significance of differences in the treatment effect was calculated using Benjiamini–Hochberg correction.

The data were presented as a median and 25 and 75% quartiles. Statistically significant differences were considered at *p* < 0.05.

### Sample size statistical analysis

The required sample size for the patient group was estimated with an assumption of a 4-point gain in FMMA score for the BCI group compared to control group, and a standard deviation of 4.2 points based on the data from our previous experience with the scale. The sample size for each group was found to be at least 19 subjects to achieve statistical power of 80%.

## Results

### Clinical efficacy

An improvement in the upper extremity motor function was observed in both study groups as assessed by the ARAT and FMMA scores. For the BCI group, we found improvements in the grasp from 0.0 [0.0; 14.0] to 3.0 [0.0; 15.0] points (*p* < 0.01), pinch from 0.0 [0.0; 7.0] to 1.0 [0.0; 12.0] points (*p* < 0.01), and gross movements from 2.0 [0.0; 5.0] to 3.0 [0.0; 7.0] points (*p* < 0.01) (ARAT score; Table 3). For the control group, grasp, pinch and gross movement scores did not improve (*p* > 0.05).

**Table 3:** Efficacy measures by ARAT and FMMA scores for each study group (all randomized patients).

**Outcome**		**BCI (*n* = 55)**	**Control (*n* = 19)**	**Possible range**
ARAT total	Before	4.0 [0.0; 31.0]	3.0 [0.0; 30.0]	0–57
	After	6.0 [1.0; 43.0]	6.0 [0.0; 31.0]	
	*p*	<0.01	0.021	
ARAT-Grasp	Before	0.0 [0.0; 14.0]	0.0 [0.0; 12.0]	0–18
	After	3.0 [0.0; 15.0]	1.0 [0.0; 12.0]	
	*p*	<0.01	0.394	
ARAT-Grip	Before	0.0 [0.0; 8.0]	0.0 [0.0; 6.0]	0–12
	After	1.0 [0.0; 10.0]	1.0 [0.0; 7.0]	
	*p*	<0.01	0.045	
ARAT-Pinch	Before	0.0 [0.0; 7.0]	0.0 [0.0; 4.0]	0–18
	After	1.0 [0.0; 12.0]	0.0 [0.0; 5.0]	
	*p*	<0.01	0.675	
ARAT-Gross movement	Before	2.0 [0.0; 5.0]	2.0 [0.0; 6.0]	0–9
	After	3.0 [0.0; 7.0]	3.0 [0.0; 6.0]	
	*p*	<0.01	0.273	
FMMA upper extremity	Before	24.0 [12.0; 40.0]	12.0 [11.0; 49.0]	0–66
	After	29.0 [14.0; 47.0]	17.0 [12.0; 51.0]	
	*p*	<0.01	<0.01	
FMMA-Proximal	Before	20.0 [10.0; 27.0]	11.0 [9.0; 27.0]	0–42
	After	24.0 [13.0; 32.0]	15.0 [11.0; 28.0]	
	*p*	<0.01	<0.01	
FMMA- Distal	Before	5.0 [1.0; 14.0]	2.0 [1.0; 16.0]	0–24
	After	7.0 [2.0; 18.0]	3.0 [1.0; 16.0]	
	*p*	<0.01	0.046	

*Medians and 25 and 75% quartiles are shown. The values of p < 0.05 are in red (it means statistically significant difference)*.

**Figure 3:**
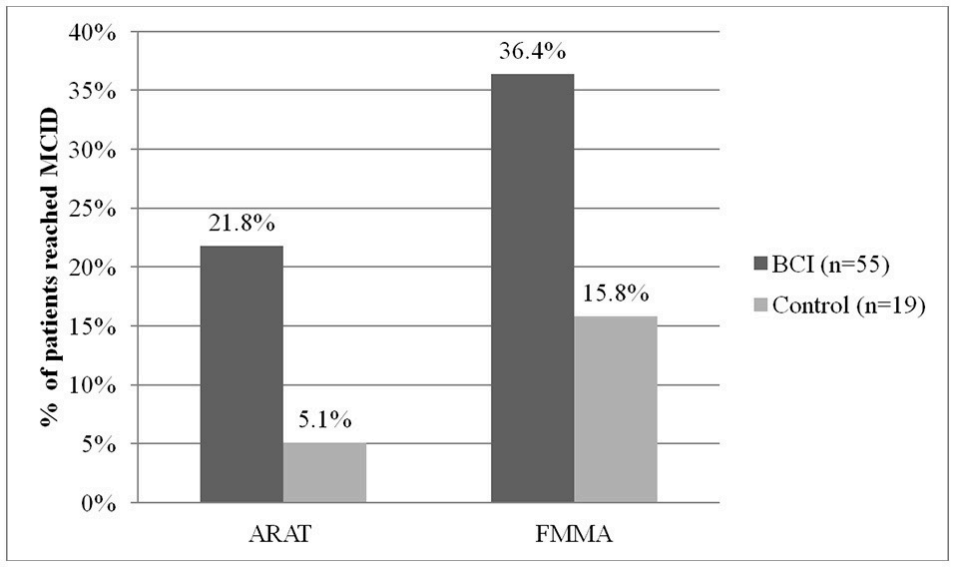
Percent of patients reached minimal clinically important difference (MCID) by ARAT and FMMA scores in each study group.

In the BCI group, the percentage of patients who achieved MCID was 4.3 times higher than in the control group in the ARAT and 2.3 times in the FMMA (Figure 3). The observed positive changes in the BCI group were mainly due to recovery of the hand motor function.

More outcomes improved significantly (*p* < 0.05 compared to baseline) in BCI-trained patients with subacute (Table 4) and chronic (Table 5) stroke. For both subacute (Table 4) and chronic (Table 5) cohorts the percentage of patients who achieved MCID was higher in the BCI group.

**Table 4:** Efficacy measures by ARAT and FMMA scores in subacute stroke patients (1–6 months from onset) for each study group.

**Outcome**		**BCI (*n* = 25)**	**Control (*n* = 9)**	**Possible range**
ARAT total	Before	1.0 [0.0; 15.0]	13.0 [0.0; 22.0]	0–57
	After	3.0 [0.0; 21.0]	12.0 [0.0; 24.0]	
	*p*	<0.01	0.150	
ARAT-Grasp	Before	0.0 [0.0; 7.0]	4.0 [0.0; 7.0]	0–18
	After	0.0 [0.0; 10.0]	4.0 [0.0; 6.0]	
	*p*	0.036	0.552	
ARAT-Grip	Before	0.0 [0.0; 4.0]	2.0 [0.0; 5.0]	0–12
	After	0.0 [0.0; 6.0]	3.0 [0.0; 7.0]	
	*p*	0.054	0.181	
ARAT-Pinch	Before	0.0 [0.0; 1.0]	2.0 [0.0; 4.0]	0–18
	After	0.0 [0.0; 2.0]	2.0 [0.0; 3.0]	
	*p*	0.029	0.593	
ARAT-Gross movement	Before	1.0 [0.0; 4.0]	2.0 [0.0; 5.0]	0–9
	After	1.0 [0.0; 5.0]	3.0 [0.0; 6.0]	
	*p*	0.086	0.564	
FMMA upper extremity	Before	15.0 [6.0; 36.0]	12.0 [11.0; 29.0]	0–66
	After	18.0 [13.0; 46.0]	17.0 [12.0; 31.0]	
	*p*	<0.01	0.144	
FMMA-Proximal	Before	12.0 [7.0; 26.0]	11.0 [10.0; 20.0]	0–42
	After	16.0 [12.0; 29.0]	15.0 [11.0; 23.0]	
	*p*	<0.01	0.112	
FMMA- Distal	Before	2.0 [1.0; 9.0]	1.0 [1.0; 9.0]	0–24
	After	3.0 [1.0; 12.0]	4.0 [1.0; 8.0]	
	*p*	0.028	518	
Cases with MCID (ARAT), % (n)	8.0 (2)	0 (0)	0–100	
Cases with MCID (FMMA), % (n)	24.0 (6)	11.1 (1)	0–100	

*The values of p < 0.05 are in red (it means statistically significant difference)*.

**Table 5:** Efficacy measures by ARAT and FMMA scores in chronic stroke patients (>6 months from onset) for each study group.

**Outcome**		**BCI (*n* = 30)**	**Control (*n* = 10)**	**Possible range**
ARAT total	Before	18.5 [1.0; 39.0]	2.0 [0.0; 30.0]	0–57
	After	27.0 [3.0; 45.0]	2.0 [1.0; 37.0]	
	*p*	<0.01	0.086	
ARAT-Grasp	Before	8.0 [0.0; 17.0]	0.0 [0.0; 12.0]	0–18
	After	10.0 [0.0; 18.0]	0.0 [0.0; 12.0]	
	*p*	<0.01	1.0	
ARAT-Grip	Before	4.5 [0.0; 9.0]	0.0 [0.0; 8.0]	0–12
	After	6.5 [0.0; 10.0]	0.0 [0.0; 8.0]	
	*p*	<0.01	1.0	
ARAT-Pinch	Before	2.0 [0.0; 10.0]	0.0 [0.0; 6.0]	0–18
	After	4.0 [0.0; 12.0]	0.0 [0.0; 8.0]	
	*p*	<0.01	0.678	
ARAT-Gross movement	Before	2.0 [1.0; 6.0]	2.0 [0.0; 6.0]	0–9
	After	3.0 [1.0; 8.0]	2.0 [1.0; 6.0]	
	*p*	<0.01	1.0	
FMMA upper extremity	Before	30.5 [17.0; 41.0]	12.5 [11.0; 49.0]	0–66
	After	38.0 [19.0; 53.0]	15.5 [13.0; 56.0]	
	*p*	<0.01	0.096	
FMMA-Proximal	Before	22.0 [15.0; 29.0]	11.5 [9.0; 27.0]	0–42
	After	26.0 [14.0; 32.0]	14.0 [10.0; 28.0]	
	*p*	<0.01	0.075	
FMMA- Distal	Before	8.0 [2.0; 15.0]	2.5 [1.0; 19.0]	0–24
	After	13.0 [3.0; 19.0]	3.0 [2.0; 22.0]	
	*p*	<0.01	0.072	
Cases with MCID (ARAT), % (n)	33.3 (10)	10.0 (1)	0–100	
Cases with MCID (FMMA), % (n)	46.7 (14)	20.0 (2)	0–100	

*The values of p < 0.05 are in red (it means statistically significant difference)*.

Neither in the BCI group, nor in the control group was upper extremity function recovery (according to the ARAT and FMMA scores and subscores) correlated with post-stroke duration and patient age. In each group, a moderate positive correlation was found between the extent of upper extremity function recovery (in particular, hand function recovery) and initial severity of a neurological deficit (*p* < 0.05).

### BCI accuracy

Both maximum and mean (of all trials) values in which motor imagery-related EEG activity was detected, were higher than chance level and reached 51.9 [45.0; 65.0]% and 40.6 [36.8; 46.5]%, respectively. The individual maximal detection level was achieved across different patients at different time points: from the first to ninth training day. We found a correlation between the best classification accuracy achieved and the improvement in the upper extremity function (*r* = 0.42, *p* = 0.014; ARAT score). Additionally, we found a correlation between the mean classification accuracy rate and the improvement in the upper extremity function (*r* = 0.52, *p* = 0.002 with ARAT score and *r* = 0.35, *p* = 0.04 with FMMA score).

### Safety

According to the ARAT and FMMA scores, no significant deterioration in the upper extremity function was observed in the patients during the study.

During training sessions, 3 patients developed a slight headache. Those were 2 patients from the BCI group (this symptom was observed during 2 of 10 trainings in one of them and in the course of 10 training sessions in the second patient) and 1 patient from the control group (in 3 of 10 training sessions).

The majority of patients reported fatigue associated with concentration of attention after about 20–30 min of training. The fatigue was more pronounced when patients experienced insomnia during the night preceding training (2 patients of the BCI group), suffered from symptoms of depression (2 patients of the BCI group), underwent other rehabilitation procedures prior to training (1 patient of the BCI group), or experienced general weakness. Most patients believed that the sensation of fatigue was related to training intensity, but were willing to accept some fatigue to achieve functional improvement.

Upon a complaint of headache or tiredness, the training was canceled on that day. In one patient from the BCI group who developed fatigue, the interval between trial blocks within the same training session was increased to 2–3 min (based on a medical doctor's permission and the patient's request). In one patient, the interval between training days was increased to 2–3 days due to fatigue and a poor general condition.

Increased blood pressure of 200/100 mm Hg occurred in one patient from the BCI group after the third training day. This issue was therapeutically reversed.

Overall, none of the patients withdrew from the study because of adverse events.

## Discussion

Our multicenter, blind, controlled study showed that a repeated BCI training in which motor imagery-related EEG activity is translated into contingent movements of a hand exoskeleton can have a positive effect on motor function in post-stroke patients. This improvement in motor function was reflected by the percentage of patients showing clinically significant functional recovery of upper extremity motor function as measured by MCID. The positive effect of BCI training was predominantly due to recovery in hand function, which was the body part imagined moving during the BCI training. Moreover, an improvement of grasp and pinch was observed only in the BCI-exoskeleton group. This result could be partially explained by the insignificantly greater initial severity of impairment in the control group, because the greater initial impairment predicts the worse functional recovery (Stinear, 2010; Coupar et al., 2012). However, this explanation could be true only for chronic patients having greater initial impairments in the control group comparing to BCI group (Table 2). For subacute patients it was opposite: for control group the initial impairments were less than in the BCI group, however, the recovery in grasp, pinch and gross movements was worse. It is important to note that grasping a large object (e.g., special objects used for the assessment by the ARAT score) required performing a hand opening movement, which was the movement patients imagined during the BCI sessions. Nevertheless, according to ARAT and FMMA total scores no significant differences between the BCI and control groups in the extent of motor function recovery were found (Table 3), which may have been due to an insufficient duration of trainings compared with other studies (Ang et al., 2015).

The results of our study are consistent with the results of the previous controlled studies that employed motor-imagery BCIs. In particular, Ramos–Murguialday et al. reported a controlled trial with 32 chronic post-stroke patients: 16 patients trained with a motor imagery-based BCI with hand and arm orthosis feedback coupled with physiotherapy and 16 patients received sham orthosis feedback and physiotherapy alone. Training in both groups continued for 4 weeks, except weekends (patients underwent about 18 training sessions, on average). An improvement of the motor function in the BCI group, as assessed with FMMA, was 3.41 points higher, on average, than that in the control group (*p* = 0.018; Ramos-Murguialday et al., 2013).

In a study by Ang et al. which involved 26 subacute and chronic post stroke patients, the treatment effect was compared between a group of subjects who received training with BCI-controlled MIT-Manus and a group of subjects treated only with the MIT-Manus robotic device. In this case, the training intensity in the MIT-Manus group was much higher compared to that in the BCI-Manus group (1,040 vs. 136 movements per session, respectively). The treatment efficacy was comparable in both groups after 4 weeks of training. But 12 weeks after study beginning, a further improvement of upper extremity motor functions was observed in 63.6% of BCI-Manus group patients compared to 35.7% of control group patients (Ang et al., 2015). Another study by Ang et al. involving 21 patients with post-stroke duration of more than 4 months compared three approaches: BCI with a Haptic Knob robotic device for hand opening (BCI-HK group), the Haptic Knob device without BCI control, and a standard rehabilitation program (Ang et al., 2014). A significantly greater improvement of the hand function compared to standard therapy was achieved only in the BCI-HK group. Such improvements were observed on the 3rd, 12th, and 24th weeks of the study (2.14, 1.82, and 2.28 points, respectively; FMMA scale, *p* < 0.05).

It should be noted that, in contrast to our study, patients in the described studies were pre-screened for the ability to operate motor imagery-based BCI. Our inclusion criteria did not imply this pre-screening and we succeeded to assess the dependence of motor improvements on the ability to elicit motor imagery-related EEG modulations. Another important difference of those studies is a much greater intensity of trainings: 18 h for the entire course (Ang et al., 2014, 2015) compared to our study where the overall training time was 5 h, on average. However in spite of the short training time we obtained significant differences between the main and control groups in grasp and pinch ARAT scores.

Contrary to other studies with almost equal number of patients in BCI and control groups our control group constitutes only one third of the BCI group that reduces a statistical power of the study. Nevertheless the control group in our study (19 patients) was more numerous than in others studies of this kind—16 in Ramos-Murguialday et al. (2013), 15 in Ang et al. (2015), 8 in Ang et al. (2014), and 6 in Ono et al. (2014). Non-parametric methods used in our analysis are able to reveal statistical differences between the two groups.

Our study also demonstrated that recovery of the upper extremity function (according to ARAT and FMMA scores and subscores) in the BCI was observed in both subacute and chronic subgroups of patients. Therefore, BCI training may be indicated for patients in both rehabilitation periods and may promote better recovery, which is also consistent with the other studies (Buch et al., 2012; Ang et al., 2014, 2015). This suggestion is supported by a recent systematic review that recommended motor imagery for rehabilitation of motor function in subacute and chronic stroke as an “adjuvant therapy” (Hatem et al., 2016). In the case of motor imagery-based BCI combined with the exoskeleton, this kind of intervention is enhanced by kinesthetic feedback. Moreover, Ono et al. demonstrated in a small pilot trial that visuo-kinesthetic feedback provides benefits compared to pure visual feedback for motor imagery -based BCI training in post-stroke subjects (Ono et al., 2014).

In our study, all subjects with subacute or chronic focal brain damage and hand paresis were able to operate the motor imagery-based BCI. For quantitative assessment of this ability, the average of percentages of the correctly classified trials of paretic hand imagery, unaffected hand imagery and resting state was used as an index of BCI accuracy and, at the same time, of the ability to elicit motor imagery-related EEG signal modulations. The maximum and mean classification accuracy rates were higher than chance level. This quantitative assessment is consistent with the results of other studies showing that patients with focal brain damages, similar to healthy people, can operate a BCI based on motor imagery (Buch et al., 2012; Ang et al., 2014, 2015; Frolov et al., 2017).

The kinesthetic imagination of both affected and unaffected limbs and even transition to the motor relaxation are related to motor functions and generally influence the mechanisms of neuroplasticity resulting in motor recovery. Thus, it is reasonable to suppose that motor recovery depends on reorganization of brain activity in both damaged and not damaged hemispheres. Particularly, this assumption seems correct in the case of extensive brain lesions when the resource of the damaged (ipsilesional) hemisphere is insufficient (Mokienko et al., 2016). Therefore, the successful performance of all three mental tasks is important for motor recovery and the reported index of BCI accuracy seems to be an adequate predictor of motor improvement. The correlation between motor improvements and BCI accuracy confirmed this hypothesis. Our results show that an improvement of motor function had taken place for all patients included in the study independently of duration, severity and location of the stroke. Thus, the ability to control a motor imagery-based BCI could be considered as a key to identify patients with the greatest rehabilitation potential.

One of the methodological features of this study is the use of 2 scales for assessing recovery of upper extremity motor function. The FMMA scale is a more versatile and detailed one (Sanford et al., 1993; Ang et al., 2014), while ARAT is a functional scale and evaluates different hand movements needed for daily tasks (Doussoulin et al., 2012).

The study design incorporated the recruitment of patients from three clinical centers. Testing of patients by specialists from different clinical centers and applying a blind study design was implemented to reduce the influence of subjective factors (Sanford et al., 1993) on the assessment of clinical test performance.

Also, an important difference to other studies in this area is that an exoskeleton-mediated movement was kinematically closer to a physiological movement of the hand and each finger. This is mainly achieved through the use of flexible “pneumatic muscles,” “exo-joints,” and finger fixators respecting the anatomical structure of the human hand, which improves ergonomics, does not lead to rapid fatigue of the patient during use of the system, and eliminates the risk of injuries.

The most common adverse event was fatigue, but none of the patients withdrew from the study due to a serious adverse event, which suggests that the technology is safe in general. Since fatigue cases were worsened by insomnia, large exercise load of preceding procedures, propensity to depression, and by general weakness, the likelihood of fatigue can be reduced by selecting the optimal sequence of rehabilitation procedures and surveying the patients in regard to their current state of health and quality of sleep before each training session.

### Study limitations

The main limitation of the reported study is the low number of training sessions and lack of follow-up assessments after the training course was completed. The overall training time was 5 h, on average. However, we could not increase the intensity of training within the study framework due to the center rules and limited hospital stay.

An additional limitation of the study is that study groups had different sample sizes (55 vs. 19). However, statistical analyses were chosen to compensate for this difference in sample sizes. It should be noted, however, that the sample size of the subgroups (subacute and chronic) within each study group was under-powered. Due to the small sample size and heterogeneity within subjects, the results of the subgroup analysis need to be interpreted with great caution.

### Future research direction

Future studies with larger sample sizes are needed to corroborate the here reported findings. To determine patients who will most likely benefit from motor imagery-based BCI training, it will be important to identify neuropsychological, physiological, and clinical factors that predict BCI treatment response.

## Ethics statement

The study protocol was conducted in accordance with the Helsinki Declaration and was approved by the Ethical Committee of the Research Center of Neurology (#12/14 of 10.12.2014). All patients provided written informed consent for participation in the study. The study protocol was registered in clinicaltrials.gov (“iMove,” trial number NCT02325947).

## Author contributions

All authors: Substantial contributions to the conception or design of the work; or the acquisition, analysis, or interpretation of data for the work; Drafting the work or revising it critically for important intellectual content; Final approval of the version to be published; Agreement to be accountable for all aspects of the work in ensuring that questions related to the accuracy or integrity of any part of the work are appropriately investigated and resolved.

### Conflict of interest statement

The authors declare that the research was conducted in the absence of any commercial or financial relationships that could be construed as a potential conflict of interest.
